# Role of Nrf2 and Its Activators in Cardiocerebral Vascular Disease

**DOI:** 10.1155/2020/4683943

**Published:** 2020-08-05

**Authors:** Liangkai Cheng, Hong Zhang, Fang Wu, Zhongqiu Liu, Yuanyuan Cheng, Caiyan Wang

**Affiliations:** ^1^International Institute for Translational Chinese Medicine, Guangzhou University of Chinese Medicine, Guangzhou, Guangdong 510006, China; ^2^Shunde Hospital of Guangzhou University of Chinese Medicine, Shunde, Guangdong 528300, China

## Abstract

Cardiocerebral vascular disease (CCVD) is a common disease with high morbidity, disability, and mortality. Oxidative stress (OS) is closely related to the progression of CCVD. Abnormal redox regulation leads to OS and overproduction of reactive oxygen species (ROS), which can cause biomolecular and cellular damage. The Nrf2/antioxidant response element (ARE) signaling pathway is one of the most important defense systems against exogenous and endogenous OS injury, and Nrf2 is regarded as a vital pharmacological target. The complexity of the CCVD pathological process and the current difficulties in conducting clinical trials have hindered the development of therapeutic drugs. Furthermore, little is known about the role of the Nrf2/ARE signaling pathway in CCVD. Clarifying the role of the Nrf2/ARE signaling pathway in CCVD can provide new ideas for drug design. This review details the recent advancements in the regulation of the Nrf2/ARE system and its role and activators in common CCVD development.

## 1. Introduction

Cardiocerebral vascular disease (CCVD) is a common disease that seriously threatens human health, especially in people over 60 years old [1]. Great efforts are being made to develop effective therapies for the prevention and treatment of CCVD to improve human health. Hyperlipidemia, hypertension, and increased of blood viscosity are the main causes of CCVD [2–4]. Although the pathogenesis of CCVD is complicated and varied, an increasing number of studies have implicated that oxidative stress (OS) is involved in CCVD. Once OS produces excessive oxidative intermediates, such as reactive oxygen species (ROS) and reactive nitrogen species (RNS), tissue damage and even organ injury follow [5]. The main endogenous ROS is derived from xanthine oxidase, mitochondria, nicotinamide adenine dinucleotide phosphate (NADPH) oxidase, lipoxygenase, uncoupling of nitric oxide synthase (NOS), and so on [6, 7]. ROS mediate multiple signaling pathways that promote vascular inflammation in the atherogenic process and causes vascular cell dysfunction [8–10]. Additionally, OS and excessive production of ROS can exacerbate myocardial or neurological dysfunction in CCVD [11].

The Nrf2/antioxidant response element (ARE) pathway governs the gene expression of endogenous antioxidant synthesis and ROS-eliminating enzymes and can prevent cellular damage caused by OS and ROS [12]. Nrf2-driven antioxidant defense system also regulates a large number of detoxification and antioxidant enzymes and thus plays an important role in CCVD [13, 14]. The activation of Nrf2 can ameliorate cardiac dysfunction and has obvious protective effects on the heart and blood vessels, thereby preventing and delaying cardiovascular disease (CVD). Moreover, the activation of Nrf2 has protective and preventive effects on cerebrovascular damage and neurological dysfunction [15]. Therefore, treatment strategies that are aimed at the Nrf2/ARE signaling pathway are likely to resolve the impasse in CCVD treatment.

In this review, we focus on the Nrf2/ARE antioxidant defense system and investigate its crucial role in various CCVD pathological processes. In addition, the therapeutic effects of CCVD realized by activating the Nrf2/ARE signaling pathway reported in the literature are summarized and discussed.

## 2. Structural Characteristics and Biological Features of Keap1/Nrf2

When OS occurs, the Kelch-like ECH-associated protein 1 (Keap1)/Nrf2 signaling pathway is activated and regulates the expression of multiple antioxidants and detoxification enzymes to maintain intracellular homeostasis. Antioxidant enzymes can regulate the redox imbalance of cells and detoxifying enzymes can clear carcinogens and toxins.

**Figure 1:**
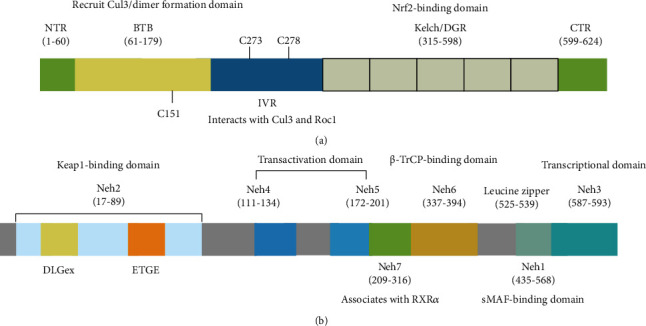
Schematic structure of Nrf2 and Keap1. (a) Domain structure of Keap1. The BTB domain recruits Cul3 and mediates Keap1 homodimerization. The IVR interacts with Cul3 and Roc1. The DGR/Kelch domain binds with Nrf2. C151, C278, and C273 are OS-sensitive cysteines. (b) Domain structure of Nrf2. The Nrf2 protein consists of 7 domains. Neh1-Neh7: Neh2 binding with Keap1 through the DLG and ETGE motifs. The binding affinity of the ETGE motif for Keap1 is 200-fold higher than that of the DLG motif. Neh4 and Neh5 bind with CREB/SRC3, facilitating Nrf2 transcription. Neh7 interacts with the retinoid X receptor to repress Nrf2. Neh6 binds *β*-TrCP to promote the ubiquitination and proteasomal degradation of Nrf2. Neh3 interacts with the transcription coactivator CHD6.

Under basal conditions, Nrf2 is continuously ubiquitinated and degraded by binding with Keap1/Cul3 ubiquitin ligase complex [16, 17]. Keap1 is a cysteine-rich, zinc-finger homodimer that contains 624 amino acids and is mainly distributed on the cytoplasmic actin cytoskeleton [16]. It is composed of an N-terminal region (NTR), BTB (bric-a-brac, tramtrack, and broad complex) domain, double glycine repeat (DGR) domain, intervening region (IVR), and C-terminal region (CTR) (Figure 1(a)). Keap1 recruits Cul3 through the BTB domain, and the BTB mediates the homodimerization of Keap1, which is necessary to sequester Nrf2 in the cytoplasm [18, 19]. The DGR domain, also known as the Kelch domain, binds with the Neh2 domain (DLGex and ETGE motif) of Nrf2. However, the binding is competitively inhibited by proteins that have ETGE-like motifs such as p62 [20]. IVR is characterized by cysteine-rich residues, which senses electrophiles and OS. In addition, the BTB-IVR domain binds to Cul3 and Roc1 to form a Keap1-Nrf2-Cul3-Roc1 complex, which helps Nrf2 to undergo proteasomal degradation [19]. The Keap1-dependent Nrf2 activation depends on the redox-sensitive reactive cysteine residues in Keap1. C273, C151, and C288, the three well-characterized cysteine residues, are the main cysteine sensors for disrupting the Keap1-Nrf2 complex [21, 22]. Other Keap1 cysteine residues, including C226, C434, and C613, seem important for sensing specific electrophiles.

Nrf2 is a transcription factor containing 605 amino acids that were originally cloned from the human leukemia cell line K562 and belongs to the cap-n-collar (CNC) family of basic leucine zippers [23]. It is composed of seven highly conserved domains named Neh1-Neh7, and each domain possesses a different function (Figure 1(b)). The Neh1 domain contains a zipper structure called CNC-bZIP and forms a heterodimer with small musculoaponeurotic fibrosarcoma (sMaf) responsible for Nrf2 binding to ARE [24]. The N-terminal Neh2 domain is the regulatory domain of Nrf2 for its interaction with Keap1. The Neh2 has a high affinity binding site to Keap1 named the ETGE motif and a low affinity site called DLGex motif, which form the structural basis of the “latch and hinge” together [25, 26]. Deletion or mutation of the ETGE or DLG motif will disrupt the Nrf2-Keap1 interaction and lead to the activation of the Nrf2/ARE pathway [26, 27]. The C-terminal Neh3 domain interacts with chromo-ATPase/helicase DNA-binding protein 6 (CHD6), which is essential for the transactivation of ARE-dependent genes after chromatin remodeling [28]. The Neh4 and Neh5 are responsible for the transcriptional activation of Nrf2 by binding with the coactivator cyclic adenosine monophosphate (cAMP) response element-binding protein (CREB) and/or steroid receptor coactivator 3 (SRC3) [29]. The Neh6 domain plays a role in phosphorylation- and ubiquitination-based regulation of Nrf2 activity. The DSGIS and DSAPGS modules of Neh6 interact with glycogen synthase kinase-3*β* (GSK-3*β*) and *β*-transducin repeat-containing protein (*β*-TrCP) to recruit the kinase-associated protein or Roc1 core E3 ubiquitin ligase to mediate Nrf2 degradation [30]. The Neh7 domain can suppress Nrf2 by interacting with retinoid X receptor [31].

## 3. Nrf2 Signaling Pathway and Antioxidative Mechanism

Nrf2 is an important transcription factor that plays a crucial role in cytoprotection against OS and regulated by multiple signaling pathways.

### 3.1. Keap1/Nrf2/ARE Signaling Pathway

**Figure 2:**
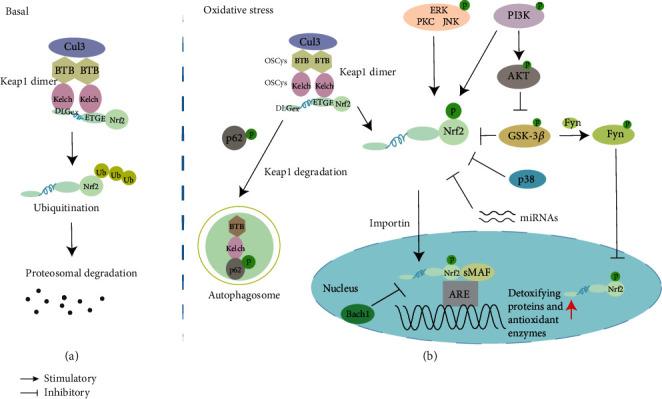
Stress response regulatory network of Nrf2. (a) Under basal conditions, Keapl is associated with Cul3 to form a homodimer through BTB/BTB domain interactions. Each Kelch/DGR domain interacts with the DLG and ETGE motifs of Nrf2. Nrf2 is polyubiquitinated and degraded by the proteasome through formation of a Cul3-Keap1 ligase complex. Ub represents ubiquitination. (b) To respond to OS, the key cysteine residues in Keap1 sense OS and disrupt the interaction between the DLGex and Kelch domains, thereby releasing Nrf2. Free Nrf2 transfers to the nucleus and forms a heterodimer with the sMaf protein, subsequently binding to the ARE and initiating the transcription of downstream antioxidant and detoxifying enzyme genes. Kinases PI3K, PKC, JNK, and ERK activate Nrf2 by phosphorylation. GSK-3*β* can promote Nrf2 translocation from the nucleus through Fyn kinase activation and lead to the deactivation of Nrf2 in the nucleus. p38-MAPK and GSK-3*β* inhibit Nrf2 activation. OS regulates p62 through phosphorylation, and phosphorylated p62 binds to Keap1, resulting in the autophagic degradation of Keap1. Under normal conditions, Bach1 (BTB and CNC homology 1) forms a heterodimer with the sMaf protein and thereby suppresses Nrf2 activation. miRNAs, such as miR-27a, miR-142-5p, and miR-153, can affect the level of Nrf2 protein in a Keap1-independent manner. p: phosphorylation.

The Keap1/Nrf2/ARE signaling pathway is the most critical pathway to regulate the Nrf2 and cellular antioxidant stress defense system. At the homeostatic state, Nrf2 binds with Keap1 and is degraded through the ubiquitin proteasome pathway (Figure 2(a)). When subjected to OS and electrophilic, the key cysteine residues on Keap1 are covalently modified, leading to a conformational change of Keap1 [32–34], following the disassociation of Keap1 from the DLG motif, and therefore, Nrf2 is activated (Figure 2(b)). Subsequently, the free Nrf2 enters the nucleus and binds to ARE to initiate the expression of downstream phase II gene detoxification enzymes and antioxidant enzymes, such as heme oxygenase-1 (HO-1), NAD(P)H dehydrogenase, quinone 1 (NQO1), glutathione-S-transferase (GST), superoxide dismutase (SOD), aldehyde dehydrogenase-1,2, UDP-glucuronosyltransferase, glutamate-cysteine ligase, and regulatory subunits including thioredoxin reductase 1, sulfiredoxin, glutathione reductase (GR), peroxiredoxin (Prx), thioredoxin (Trx), catalase, and glutathione peroxidase (Table 1).

p62/SQSTM1 is an autophagy adaptor protein that was initially identified as a tyrosine kinase p56lck-binding protein. The 349-DPSTGE-354 motif (STGE motif), located in the Keap1-interacting region (KIR) domain of p62, resembles the ETGE motif in the Neh2 domain of Nrf2 which binds with Keap1 and the initial autophagic degradation of Keap1; subsequently, Nrf2 is released to activate the target genes [20, 35]. OS signaling induces p62-mediated Nrf2 activation by regulating p62 phosphorylation [36]. For example, the phosphorylation of S349 in the STGE motif can enhance the binding of Keap1 with p62, thus further activating Nrf2 [36, 37] (Figure 2(b)).

### 3.2. Other Mechanisms of Nrf2 Regulation

Nrf2 is a degradation target of synoviolin (Hrd1) and *β*-TrCP. The degradation of Nrf2 by *β*-TrCP depends on GSK-3*β*, which phosphorylates specific serine residues in the Neh6 domain of Nrf2 to create a degradation domain that is recognized by the ubiquitin ligase adapter *β*-TrCP and tagged for proteasome degradation by the Cullin1-Rbx1 complex [38]. Hrd1 is an E3 ubiquitin ligase anchored to the endoplasmic reticulum and reacts with X-Box binding protein 1, which is associated with the unfolded protein response, causing the degradation of Nrf2 (the endoplasmic reticulum-associated degradation) [39, 40]. However, considering that Keap1 has a strong effect on Nrf2, under acute oxidative conditions, *β*-TrCP/Hrd1-mediated Nrf2 degradation may not affect the activation of Nrf2 upon Keap1 oxidation. Furthermore, Hrd1 is associated with cardiac dysfunction, cardiac hypertrophy, and the apoptosis of cardiomyocytes [41]. Therefore, the study of the interaction between Hrd1 and Nrf2 during heart injury may further reveal the pathogenesis of CVD.

Moreover, protein kinases also play a vital role in regulating Nrf2. Threonine or serine accounts for approximately 15% of the amino acids in the Nrf2 protein, which are potential phosphorylation sites for threonine/serine kinases. Phosphorylation of specific residues of Nrf2 can increase its stability and transactivation activity. The typical protein kinases of phosphatidylinositol 3-kinase (PI3K), extracellular signal-regulated protein kinase (ERK), protein kinase C (PKC), and c-Jun N-terminal kinase (JNK) positively regulate Nrf2, while p38-MAPK (mitogen-activated protein kinases) feedback negatively regulates the Nrf2 pathway [42–44]. The effect of the AKT kinase is associated with modulation of GSK-3*β*, which can directly phosphorylate Nrf2 protein at a serine in the DSGIS motif in the Neh6 domain [30, 45]. In addition, GSK-3*β* can negatively regulate the Nrf2 by activating Fyn kinase (cause Nrf2 inactivation and away from the nucleus) [46] (Figure 2(b)).

MicroRNAs (miRNAs) are regulators that control protein production by inducing posttranscriptional and/or translational inhibition [47]. Nrf2 activity is also regulated by miRNAs at the posttranscriptional level. Specifically, miR-27a, miR-142-5p, miR-144, miR-153, miR-34a-5p, and miRNA-199a-5p can affect the level of Nrf2 protein [48–50]. Additionally, under normal conditions, the Bach1 (BTB and CNC homology 1) and sMaf proteins form a heterodimer to suppress the activation of Nrf2 [51] (Figure 2(b)).

## 4. Cross Talk between Nrf2 and Other Signaling Pathways

Both Nrf2 and NF-*κ*B are activated in response to OS, but they produce opposite effects on the body. In general, Nrf2-induced genes have anti-inflammatory and antioxidant effects, while NF-*κ*B promotes inflammation and OS. Abundant evidence suggests that these two opposing effects play an antagonistic or coordinating role in maintaining redox balance. Specifically, when activated Nrf2 is elevated, downstream genes regulated by Nrf2 will create a reducing environment to limit the activity of NF-*κ*B [52]. Conversely, inactivation of Nrf2 reduces NF-*κ*B suppression, thereby exacerbating the inflammatory response [53]. Actually, the interaction of Nrf2 and NF-*κ*B differs by cell types and tissue context due to the extensiveness of the genes induced by these two transcription factors [53, 54]. Determining the specific correlation between the Nrf2 and the NF-*κ*B cross talk networks helps to further clarify the anti-inflammatory mechanisms of Nrf2 activators in CCVD.

In addition to NF-*κ*B, Nrf2 also cross talks with the Notch signaling pathway. The Notch signaling pathway can regulate cell proliferation, differentiation, wound healing, and homeostasis through cell-cell communication [55]. Notch directly induces expression of Nrf2 and its target genes via the recruitment of the Notch intracellular domain transcriptome to a conserved Rbpjk site in the promoter of Nrf2 [56]. Therefore, Nrf2 plays an important role in tissue repair and homeostasis through cross talk with the Notch signaling pathway. For example, ROS can regulate stem cell homeostasis through Nrf2-dependent Notch signaling [57]. The endothelial progenitor cells could exert therapeutic effect on acute lung injury via miR-141-3p-Notch-Nrf2 axis [58]. Furthermore, through overlapping transcriptional targets, Nrf2 cross talks with heat shock factor 1 to exhibit cytoprotection [59].

## 5. Nrf2 Signaling Pathway and CVD

Inflammatory factors and ROS produced by OS play key roles in the deterioration of CVD. Excessive production of ROS or RNS promotes the formation of hypertension and impairs the processing of cardiac calcium, resulting in arrhythmias and enhancing cardiac remodeling [60]. Moreover, OS is related to the development of cardiac dysfunction and the pathogenesis of heart failure caused by myocardial cell apoptosis [61]. The complex network of antioxidant enzymes and detoxifying enzymes regulated by Nrf2 shows that Nrf2 is a “golden finger” in preventing CVD damage [62]. For instance, during cardiac hypertrophy and myocardial ischemia-reperfusion injury (MIRI), the activation of Nrf2 has been proven to protect cardiomyocytes, thereby reducing myocardial infarct size [63]. NADPH oxidase-4 can activate the Nrf2 signaling pathway to regulate the redox of glutathione (GSH) in myocardial cells and protect the hearts of mice against chronic hypertension [64]. Nrf2 plays an important role in CVDs by maintaining redox homeostasis, such as hypertension, atherosclerosis, myocardial infarction (MI), and heart failure [65]. In addition, Nrf2 knockout mice provide a convenient model for Nrf2 research into CVDs, and many Nrf2 activators have been tested, which can greatly advance the research on CVD prevention and therapy.

### 5.1. Effects of Nrf2 Activation on Hypertension

Hypertension, known as a silent killer, is the most common chronic disease worldwide. Despite advanced drug treatment strategies, it remains a major cause of death worldwide. The relationship between Nrf2 and the development of hypertension is unclear, but substantial evidence indicates that Nrf2 activation can prevent the development of hypertension. For example, maternal Nrf2 activation by dimethyl fumarate can protect male rat adult offspring against hypertension in adulthood that is triggered by a combination of dexamethasone and high-fat diet exposures [66]. In addition, the activation of Nrf2 has a positive therapeutic effect on cell and organ damage caused by hypertension. For instance, luteolin, a natural flavonoid compound, ameliorated sodium fluoride-induced hypertension and cardiovascular complications by activating the Kim-1/NF-*κ*B/Nrf2 signaling pathways [67]. A functional bioactive peptide in potato enhances the Nrf2-induced antioxidant defense system to attenuate renal damage in hypertensive rats [68]. Furthermore, the activation of Nrf2 also obviously protects the blood vessels challenged by hypertension. For instance, the overexpression of Sirtuin6 alleviated angiotensin II-induced vascular endothelial cell apoptosis and OS by promoting the Nrf2/ARE antioxidant signaling pathway [69]. Similarly, through the Nrf2 and NF-*κ*B pathways, tilapia by-product oligopeptides had a vascular protective effect on human umbilical vein endothelial cells in an angiotensin II-induced hypertension endothelial injury context [70]. In contrast, the absence or mutation of Nrf2 promotes the development of hypertension. For example, deletion of the *Nrf2* gene in the rostral ventrolateral medulla (RVLM) leads to a significant increase in the blood pressure [71]. Genetic variations decreasing the expression of *Nrf2* may affect the trigger of downstream target genes, thereby increasing the risk of hypertension and coronary atherosclerosis [72]. In addition, mutations in *Nrf2* have been shown to cause a multisystem disorder [73].

### 5.2. Effects of Nrf2 Activation on Atherosclerosis

OS and inflammation lead to endothelial cell damage, smooth muscle cell proliferation, and cholesterol lipid deposition, causing atherosclerosis. The absence of Nrf2 in macrophages leads to increased foam cell formation and exacerbates atherosclerosis [74]. In contrast, the activation of Nrf2 can suppress inflammation and alleviate atherosclerosis. In the initial stage of atherosclerosis, epigallocatechin gallate (EGCG), a polyphenol rich in green tea, can reduce the production of ROS and inhibit inflammation and endothelial cell apoptosis by activating the Nrf2/HO-1 pathway [75]. EGCG also inhibits the lipid metabolism of macrophage foam cells by activating the Nrf2/Keap1 pathway to upregulate the ATP-binding membrane cassette transporter A1 [76]. In addition, Z-ligustilide, a natural benzoquinone derivative in many widely used Chinese herbal medicines, is an effective Nrf2 activator that can protect vascular endothelial cells (VECs) against the high-fat diet-induced atherosclerosis [77]. Resveratrol, a nonflavonoid polyphenol from grapes, suppresses the expression of intercellular adhesion molecule-1 through the transcriptional regulation of the FERM-kinase (a domain of focal adhesion kinase) and Nrf2 interaction, thereby blocking monocyte adhesion and delaying the onset of atherosclerosis [78]. In addition, a novel derivative of resveratrol, *trans*-3,5,4′-trimethoxystilbene, can suppress foam cell formation to protect against atherosclerosis [79]. These findings demonstrate that the activation of Nrf2 can inhibit atherosclerosis. However, some evidence suggests that Nrf2 also exacerbate atherosclerosis. For example, Nrf2 promotes atherosclerosis by affecting plasma lipoproteins and cholesterol transport [80]. *Nrf2* knockout has been shown to protect against atherogenesis in ApoE-deficient mice [81]. In addition, atherosclerosis is exacerbated significantly by curcumin (Nrf2 activator) in ApoE-deficient mice through increasing expression of CD36 [82]. Nrf2 also contributes to cholesterol crystal-induced inflammasome activation and atherosclerosis worsening [83]. These findings implied that the relationship between Nrf2 and the development of atherosclerosis is complex.

In conclusion, Nrf2 not only inhibit the deterioration of atherosclerosis by suppressing OS, inflammation, and plaque deposition [84] but also play a negative role in atherosclerosis. This complex relationship between Nrf2 and the development of atherosclerosis indicates that the atherogenic effect of the Nrf2 activator needs to be considered when it is used clinically.

### 5.3. Effects of Nrf2 Activation on MI and MIRI

MI can cause myocardial necrosis because of persistent ischemia and hypoxia induced by blocked coronary arteries. Myocardial damage or cell apoptosis is initially caused by increased ROS upon ischemia. Nrf2-deficient mice develop heart failure more quickly after MI and have maladaptive remodeling, including cardiac hypertrophy and left ventricular dilatation [85]. This finding indicates a key role of Nrf2 in preventing the development of MI.

Statins are effective lipid lowering agents with good safety and have a beneficial effect on patients with MI, and adjuvant use can enhance the efficacy of the current treatment strategy [86]. Although the cardioprotective mechanism of statins is unclear, studies have shown that they can ameliorate heart function by activating the Nrf2 pathway. For example, after MI, the rats treated with ulinastatin, a medication for pancreatitis, showed upregulated Nrf2, HO-1, and NQO1 and downregulated NF-*κ*B, thereby slowing the progression of MI [87]. In addition, atorvastatin can reduce infarct size and improve left ventricular function [88], and rosuvastatin (a selective HMG-COA reductase inhibitor) can suppress atrial tachycardia-induced cellular remodeling [89]. Both drugs are related to Nrf2 activation. Interestingly, lithium has been found to be effective for treating MI in rats [90]. It ameliorated MI through reducing the nerve growth factor expression via the activation of the Nrf2/HO-1 pathway. Lithium is a GSK-3*β* inhibitor, indicating that the mechanism may be related to Fyn-dependent Nrf2 activation.

The therapeutic effect of potential small-molecule inducers of Nrf2 has been studied on MI mice models. The stilbenoid pterostilbene complexed with cyclodextrin can preserve left ventricular function of MI rats by increasing the activity of GST and glutaredoxin (GRx) and the expression of Nrf2 and p-GSK-3*β* [91]. Moreover, 3-n-butylphthalide (NBP), a drug used in the treatment of ischemic stroke, can inhibit OS and the inflammatory response to reduce myocardial cell death of MI rats through the AKT/Nrf2 signaling pathway [92]. Andrographolide, a labdane diterpene, can also protect against adverse cardiac remodeling of MI mice through enhancing the Nrf2 signaling pathway [93].

Reperfusion after ischemia can restore tissue and organ function and lead to the damaged structure repair. However, MIRI-induced OS in turn affects signaling pathways, leading to apoptosis, endoplasmic reticulum stress, mitochondrial dysfunction, and changes in cell migration and proliferation [94, 95], which may lead to further deterioration of MI. The Nrf2/ARE signaling pathway can resist these changes through its anti-inflammatory, antioxidant, and cell protection effects, such as ameliorate mitochondrial dysfunction [96]. In addition, Nrf2-deficient mice have increased the sensitivity to ischemic injury [97]. It means that mice lacking Nrf2 are more vulnerable to MIRI.

Ischemic preconditioning and ischemic post-conditioning are effective methods to protect myocardial tissue against MIRI [94]. Apart from these, after posttreatment with pinacidil, the expressions of Nrf2, HO-1, NQO1, and SOD1 in rat are increased, thus significantly reducing the area of MI [98]. Moreover, hyperbaric oxygen preconditioning prevents MIRI by activating the PI3K/AKT/Nrf2 antioxidant defensive system [99]. Many strategies that activate Nrf2 have therapeutic effects on MIRI. L-Carnitine inhibited the NF-*κ*B pathway and activated the Nrf2 pathway, thereby generating protective effects on MIRI [100]. In addition, dihydroquercetin, GYY4137 (a slow-releasing hydrogen sulfide donor), alpha-lipoic acid, etc., also generated protective effects on MIRI through similar mechanisms [101–103]. L-Malate provide protection against MIRI through the typical Keap1/Nrf2 pathway [104]. Additionally, some total extracts are equally effective, such as total flavonoids from *Clinopodium chinense* (Benth.) O. Ktze which can also limit MIRI and enhance cellular antioxidant defense capacity by activating the AKT/Nrf2/HO-1 signaling pathway [105].

Overall, the expression of Nrf2 is downregulated in the hearts of MI rats, leading to the reduced expression of a series of antioxidant and detoxifying enzymes regulated by Nrf2. Nrf2-deficient rats are likely to develop heart failure faster, resulting in poor cardiac remodeling. Adjuvant use of statins in the treatment of MI is a good choice. Although reperfusion can effectively ameliorate damage caused by ischemia, MIRI needs to be avoided. Postischemic treatment or ischemic preconditioning is essential to avoid the side effects of MIRI. Many strategies to activate Nrf2 have been proven to resist MI and MIRI, which means that Nrf2 activators are worth considering for the treatment of MI and reperfusion.

### 5.4. Effects of Nrf2 Activation on Heart Failure

Heart failure is the end of the development stage of heart diseases and generally is the unfavorable outcome of pathological heart hypertrophy. The activation of Nrf2 can alleviate pathological cardiac hypertrophy [106]. The study has shown that the effect is related to upregulation of the metabolic enzymes UGT1A1 and UGT1A9 through Nrf2 binding to the promoter region of *UGT1A1* or *UGT1A9* [107]. The literature on Nrf2 in experimental models of heart failure supports its cardioprotective effect, such as bardoxolone methyl which can ameliorate MI-mediated chronic heart failure by activating Nrf2 [108]. Interestingly, overexpression of Nrf2 in the RVLM attenuated sympathetic excitement in mice with coronary artery ligation-induced chronic heart failure [109]. In addition, upregulating the Nrf2 protein in the RVLM of mice with heart failure reduced the sympathetic function [110]. This finding indicated that the activation of Nrf2 is beneficial for improving sympathetic excitement in heart failure. In addition, Nrf2 has been identified as an effective therapeutic target for angiotensin II-mediated various cardiomyopathies including cardiac fibrosis, cardiac hypertrophy, and heart failure [111–113].

Arrhythmia is one of the factors that induce heart failure; the recent study has revealed that arctigenin (ATG) (an effective anti-inflammatory drug) pretreatment showed antiarrhythmia effect against MIRI in rats which is probably associated with the Nrf2 signaling pathway [114]. Another study showed that NBP can also significantly improve cardiac function (reduced cardiac fibrosis and hypertrophy) by upregulating the PI3K/AKT/Nrf2 signaling pathway [115]. Zingerone, a phenolic alkanone isolated from ginger, has been found to be effective in improving heart remodeling via activation of the eNOS/Nrf2 pathway [116]. Furthermore, the triterpenoid 2-cyano-3,12-dioxooleana-1,9-dien-28-oic acid (CDDO) and its analog DH404, sodium sulfide, and nitrite also have a certain therapeutic effect on heart hypertrophy and failure [117–120].

In general, heart dilatation or pressure overload is the main cause of heart failure. Nrf2 activator can reduce the symptoms of heart failure, such as ventricular fibrillation, ventricular tachycardia, cardiac hypertrophy, and cardiac fibrosis. In addition, Nrf2 activation can also ameliorate sympathetic excitement.

## 6. Nrf2 Signaling Pathway and Cerebrovascular Disease (CD)

CD is brain tissue damage due to disordered intracranial blood circulation. Cerebral edema and cerebral tissue damage are common severe sequelae of CDs that are associated with many pathways. Among them, ROS and inflammation are the key mediators of neurovascular dysfunction brain and tissue damage [121, 122]. In the early stages of CD, glutamate-induced excitotoxicity and OS rapidly cause brain cell death, and in the later stages, the proinflammatory and proapoptotic mediators exacerbate the deterioration of CD such as interleukin 1 and cyclooxygenase 2 [123]. The activation of Nrf2 can effectively resist the process of acute or prolonged inflammatory and apoptosis by regulating antioxidant enzyme, detoxification enzyme, and apoptotic proteins (such as Bcl-2 and Bax) [124].

### 6.1. Effects of Nrf2 Activation on Cerebral Hemorrhage

Cerebral hemorrhage refers to bleeding caused by nontraumatic rupture of cerebral parenchymal blood vessels, with a high mortality rate [125]. Currently, there is no effective treatment for cerebral hemorrhage. Mounting evidence shows that the occurrence of OS and the inflammatory response after cerebral hemorrhage are related to secondary brain damage and neurological dysfunction. Nrf2-knockout animals have poorer functional and behavioral outcomes after subarachnoid hemorrhage (SAH) [126]. In the cerebral hemorrhage, Nrf2 may protect the astrocytes and neurons against toxic damage and regulate the expression of anti-inflammatory markers and antioxidant enzymes [127–129]. The poor prognosis of intracranial hemorrhage (ICH) is caused by OS-induced secondary brain injury (SBI). For example, blood components induce overproduction of ROS and cause cytotoxicity, which in turn causes neuronal damage and neurological deficits [130]. Therefore, the inhibition of OS and inflammation is essential for reducing SBI after cerebral hemorrhage.

The activation of Nrf2 can protect the brain by improving barrier function and reducing edema and inflammation, as well as alleviating the loss of neurons and neurological deficits. Gastrodin, the main phenolic glycoside extracted from the plant root, reduced neuronal apoptosis and neurological deficits resulting from cerebral hemorrhage by regulating the Keap1/Nrf2/HO-1 pathway [131]. Simvastatin, a cholesterol-lowering medication, promoted the recovery of the nervous system and attenuated the OS and inflammatory damage caused by cerebral hemorrhage through the Nrf2/ARE signaling pathway [132]. Interestingly, monomethyl fumarate, the pharmacologically active metabolite of dimethyl fumarate, protected rats from ICH via inhibiting OS and inflammation through activating the microRNA-139/Nrf2 axis [133]. Administration of isoliquiritigenin (a flavonoid from the roots of *Glycyrrhiza glabra*) after ICH reduced early brain injury and neurological deficits, suppressing ROS and/or NF-*κ*B-mediated NLRP3 inflammasome activation by promoting the Nrf2 antioxidant pathway [129]. Isoliquiritigenin may be a potential candidate for a new treatment strategy for ICH. In addition, melatonin, *tert*-butylhydroquinone, and mononucleotides can produce neuroprotective effects against cerebral hemorrhage by activating the Nrf2 signaling pathway [134–136]. For SAH, Nrf2 has potential protective effects through the promotion of the antioxidant and anti-inflammatory responses [126]. Mangiferin, a diphenylpyrone flavonoid, and sulforaphane have been proven to reduce brain damage after ICH. Mangiferin has neuroprotective effects on early brain injury after SAH [137]. Sulforaphane can inhibit the inflammatory response and attenuate cerebral vasospasm in rats with subarachnoid hemorrhage via activating the Nrf2/ARE pathway [138].

In conclusion, the adverse effects of early cerebral hemorrhage and a series of SBIs are related to the release of toxins from cells, triggering inflammation and OS and causing the death of nerve cells. Many studies have shown that Nrf2 activators have obvious therapeutic effects on cerebral hemorrhage, which is mainly reflected in the protection of various brain cells.

### 6.2. Effects of Nrf2 Activation on Cerebral Infarction (CI) and Cerebral Ischemia/Reperfusion Injury (CI/RI)

CI is caused by insufficient blood supply to the brain and ischemic necrosis caused by ischemia and hypoxia or softening of the brain tissue, which can lead to neurological deficits. OS is one of the main causes of neuronal cell damage and death. After CI, the activation of the Nrf2/HO-1 pathway can improve redox imbalance to protect the brain from CI injury [139].

Currently, urinary kallidinogenase (a glycoprotein composed of 238 amino acids) is widely used in the treatment of CI. Recent experiments have shown that it can inhibit neuronal apoptosis and protect rats against CI by activating the Nrf2/ARE pathway [140]. Another tissue kallikrein can protect the brain against ischemic stroke by inhibiting the TLR4/NF-*κ*B signaling pathway and activating the Nrf2 signaling pathway in rats [141]. There are also some reports about traditional Chinese medicine treatment strategies for CI. For example, the salvianolate lyophilized injection, the danshen aqueous extraction, was an ideal drug for the treatment of ischemic and hypoxic injury in rats with middle cerebral artery occlusion and type 1 diabetes, and the mechanism involved the activation of the Nrf2/HO-1 signaling pathway [142]. The Huang-Lian-Jie-Du decoction is a Chinese medicine prescription that has been proven to play a protective role in the rat model of ischemic stroke via the activation of Nrf2 [143]. Andrographolide, the main ingredient of the Chinese medicine *Andrographis paniculata*, can significantly suppress free radical formation, blood-brain barrier disruption, and CI through activating the p38/Nrf2/HO-1 signaling pathway [144]. Interestingly, immune cells and bone marrow mononuclear cells (BMMCs) have been investigated as possible treatments for CI. The research has shown that it can reduce OS, apoptosis, and inflammatory responses through the PI3K/AKT/Nrf2 signaling pathway, by which they promote the secretion of nerve and vascular cytokines, improving the neurological function and reducing the infarct scope [145].

CI causes rapid and irreversible neuronal damage in the ischemic core. If the flow is restored timely, then the surrounding congested brain tissue can be rescued. However, ROS and other free radicals are also rapidly generated during reperfusion and may aggravate CI injury [146]. The Nrf2-regulated antioxidant defense system can promote blood flow recovery to protect brain and nerve tissue during reperfusion [147]. In addition, the activation of Nrf2 also protected astrocytes, thereby protecting neurons from CI/RI [148].

Chlorogenic acid, the major polyphenolic compound in coffee, has a neuroprotective effect against cerebral ischemia/reperfusion (CI/R) rats by activating the Nrf2 pathway and inducing NQO1 and HO-1 expression [149]. Furthermore, lyciumamide A from wolfberry can prevent CI/RI by activating the PKC/Nrf2/HO-1 signaling pathway, thereby ameliorating oxidative damage and attenuating neuronal apoptosis [150]. A novel biscoumarin compound, known as COM 3, can increase the survival of neurons experiencing oxygen and glucose deprivation and reduce brain infarct volumes in mice with a MCAO by changing the conformation of Keap1 and activating the Nrf2 pathway, which balances endogenous redox activity and restores mitochondrial function [151]. The extract of Ginkgo biloba has a good effect on the treatment of stroke, and recent research shows that ginkgolides and bilobalide have obvious antioxidant effects on CI/RI by activating the AKT/Nrf2 pathway *in vitro* and *in vivo* [152]. Schizandrin A (extract of Schisandra fruit) protects against CI/RI by regulating the AMPK/Nrf2 pathway [153]. In addition, uric acid, total flavonoids extracted from *Abelmoschus esculentus* L and fucoxanthin also can effectively alleviate CI/RI by activating the Nrf2/HO-1 signaling pathway [154–156].

Overall, the activation of Nrf2 can improve the poor prognosis of CI by reducing the infarct size and conferring protection effects on the nerves. CI/R is an effective treatment for CI. However, CI/RI is a nonnegligible factor in the treatment of CI, which severely limits the rehabilitation of patients. Accumulated studies have shown that the Nrf2-induced antioxidant defense system is also effective for treating CI/RI. From this perspective, the activation of Nrf2 might be a good choice for the prevention of CI/RI and improvement of the poor prognosis of CI.

## 7. Comparison of Nrf2 Activation in CCVD

**Figure 3:**
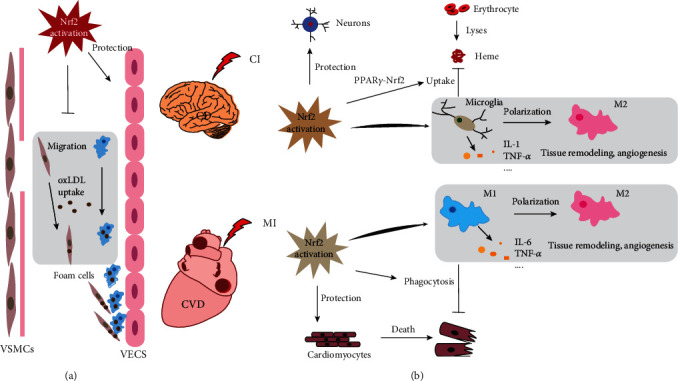
The comparison of Nrf2 activation in CCVD. (a) Nrf2 activation can protect VECs, inhibit the proliferation and migration of VSMCs, and suppress foam cell formation. OxLDL: oxidized low density lipoprotein. (b) In CI, Nrf2 activation can inhibit the activation of microglia and promote its polarization to the M2 phenotype, activate microglia uptake heme and protect neurons, and protect neurons. In MI, Nrf2 activation can promote proinflammatory M1 macrophage polarization to the M2 phenotype, enhance phagocytosis of macrophages, and protect cardiomyocytes.

Both CVD and CD are vascular system diseases, so the role of Nrf2 in CCVD is highly similar. VECs are an integral part of the heart and vasculature and play an important role in angiogenesis, hemostasis, and regulation of vascular tone [157]. Overproduction of ROS and redox imbalance are the main causes of endothelial dysfunction. Nrf2 activation can not only improve endothelial dysfunction in CCVD by reducing OS and inflammation and increasing nitric oxide bioavailability but also protect VECs by regulating the expression of antioxidant enzymes and detoxification enzymes [158]. Vascular smooth muscle cells (VSMCs) are the main structural components of the vessel wall and play a pivotal role in regulating the remodeling processes of the vessel wall. When CCVD occurs, under the stimulation of OS and inflammatory cytokines, in addition to the conversion to a calcified phenotype, the proliferation and migration of VSMCs will also be enhanced [159, 160], which may lead to intimal hyperplasia and vascular calcification. Nrf2 activation which inhibits the proliferation, migration, and calcification of VSMCs has been reported by many studies [161–163], which can ameliorate pathological changes in vascular. In addition, the activation of Nrf2 can also inhibit the formation of foam cells, including macrophages and VSMCs [164, 165], which can inhibit the production of fat streaks and are very important for maintaining vascular function (Figure 3(a)).

The role of Nrf2 also shows some differences in CCVD. Nrf2 activation can protect neurons from OS and inflammatory damage through various pathways. Microglia are professional phagocytes with immune function in the brain and play a key role in maintaining normal brain function [166], but their activation is considered to be the main cause of inflammatory damage in CI [167]. When CD occurs (such as CI), microglia are activated and release inflammatory cytokines, resulting in the inflammatory response and exacerbating brain tissue damage [168, 169]. Nrf2 activation can inhibit the activation of microglia and promote its polarization to the M2 phenotype [170, 171]. In addition, erythrocyte lysis produces heme in CCVD, which is considered to be a neurotoxin and results in brain edema and tissue damage. The PPAR*γ*-Nrf2 signaling pathway can activate the endogenous scavenging pathway, thereby promoting the removal of hematoma by microglia and ameliorating the hematoma symptoms of CI [172]. The activation of Nrf2 can also activate the expression of HO-1 to further accelerate the clearance of heme. As for CVD (such as MI), Nrf2 activation can not only protect cardiomyocytes through anti-inflammatory and antioxidant effects but also promote proinflammatory M1 macrophage polarization to the M2 phenotype, thereby ameliorating myocardial infarction injury [173]. Moreover, after MI, the timely removal of apoptotic cells is very important to restore tissue function. Nrf2 activation can enhance phagocytosis of macrophages, thereby promoting the recovery of cardiac function [174] (Figure 3(b)).

## 8. Clinical Prospects of Targeting Nrf2 for Preventing CCVD

### 8.1. The Preclinical Studies of Nrf2 Activator

Currently, many strategies have been applied for the treatment of CCVD by activating Nrf2 in the preclinical studies, which have been summarized in Table 2. Apart from commonly synthesized small molecule compounds and natural products, some novel treatments, such as elemental metals (zinc and lithium), miRNAs, cytokines, and BMMCs, are increasing.

Regarding the therapeutic effect on metal elements, lithium is commonly used in drugs for treating depression, Alzheimer's, and other neurological diseases [175]. Zinc is an essential trace element in the human body and plays an extremely crucial role in human growth and development. Zinc supplementation can protect against MIRI in rats, and lithium supplementation can significantly reduce the area of MI and protect against the adverse symptoms of MI [90, 176]. However, a determination of the therapeutic effect of zinc and lithium ion on CCVD requires more clinical data. If they are effective, then it might be a very convenient and economic treatment for patients with CCVD, as an appropriate zinc and lithium can be internalized through diet. Inhibition of miRNAs is reported to have a protective effect on CCVD by activating Nrf2. For example, the inhibition of miR-148b-3p protected neurons against OGD/R-induced apoptosis and ROS production by enhancing Sestrin2/Nrf2 antioxidant signaling [177]. Inhibition of miR-153 ameliorated ischemia reperfusion-induced cardiomyocyte apoptosis by regulating the Nrf2/HO-1 signaling pathway in rats [178]. Interestingly, dietary compounds have the potential to control atherosclerosis by targeting miR-155 to activate the Nrf2 pathway [179]. miRNAs are expected to become a new target for CCVD treatment. Recombinant cytokine drugs usually have high activity and may lead to breakthroughs in CCVD treatment. But the use of recombinant cytokine drugs is based on a series of interleukins and interferons with few current applications [180]. Epidermal growth factor can reduce the infarct size and myocardial apoptosis via activating Nrf2 [181]. Fibroblast growth factor 19 and ciliary neurotrophic factor have a good protection effect on myocardial cells by activation of the Nrf2 signaling pathway [182, 183]. These findings make the application of recombinant cytokine drugs on CCVD a possibility in the future.

### 8.2. The Usage and Clinical Trials of the Nrf2 Activator

The clinical trials and applications of the Nrf2 activators in the treatment of CCVD and other diseases are summarized in Table 3. Among them, NBP has been approved by China for the treatment of CI in 2002 [184]. It can fully treat CI clinically, significantly reduce the loss of nerve function after infarction, and improve the patient's ability to live. Dimethyl fumarate has been approved by the US FDA in 2013 to treat adult relapsing multiple sclerosis [185]. Andrographolide is an effective anti-inflammatory drug and clinically used for tonsillitis, pharyngitis, bronchitis, etc. [186]. Nrf2-related activators have been clinically tested on diabetes, kidney disease, pulmonary hypertension, bronchitis, etc., which proves their great potential as therapeutic drugs. Many studies have shown that the activation of Nrf2 can delay the deterioration of CCVD and improve the poor prognosis of CCVD, but there are very few clinical trials of the Nrf2 activator on CCVD. Therefore, the strategy of activating Nrf2 to treat CCVD is worthy of further research and development, which may break the current treatment dilemma of CCVD.

## 9. Discussion

To date, many studies have reported the impact of the Nrf2 signaling pathway on the development of CCVD [13, 126]. In CVD, the activation of Nrf2 can protect vascular tissue from OS damage and significantly attenuate cardiac arrhythmia, cardiac hypertrophy, and myocardial fibrosis. For CDs, the activation of Nrf2 can significantly alleviate cerebral nerve damage and cerebral hematoma. Therefore, reasonable regulation of Nrf2 is beneficial for the treatment and prevention of CCVD as well as improvement of its poor prognosis. A series of treatment strategies activating Nrf2 are expected to break the current deadlock in CCVD treatment.

However, most of experimental data were obtained by using Nrf2-knockout animals and under extreme conditions. In addition, experimental animals and humans have very different physiological indicators and disease tolerance, which may lead to incorrect conclusions. For example, mice and humans regulate bile acid and cholesterol metabolism differently [187], and human atherosclerotic plaques are more likely to rupture than those of mice [188]. These differences may lead to changes in the role of Nrf2 in human CCVD. It is believed that the regulatory mechanism of Nrf2 in CCVD will be more accurate and clearer in the future; the clinical treatment strategies will be more effective.

In this paper, we reviewed and discussed recent advancements in the regulatory mechanism of the Nrf2/ARE pathway and its role in CCVD. As Nrf2 can widely regulate the expression of various antioxidant enzymes and detoxifying enzymes in the body, thereby promoting anti-inflammatory and antioxidant responses, it has potential protective effects on various adverse symptoms of CCVD. Finally, various treatment strategies through Nrf2 fully demonstrate that Nrf2 is a nonnegligible target for use in CCVD treatment.

## Figures and Tables

**Table 1:** Typical downstream target proteins regulated by the Nrf2 signaling pathway.

Antioxidative or cytoprotective proteins	Function
HO-1	The rate-limiting enzyme in the process of heme catabolism; it converts heme to biliverdin.
NQO1	A flavin adenine dinucleotide-binding protein forms homodimers and reduces quinones to hydroquinones.
*γ*-Glutamylcysteine synthetase	Catalyzes and regulates GSH biosynthesis and posttranslational levels.
Glutathione peroxidase 1	An important peroxidase in the body. It can catalyze GSH into oxidized glutathione (GSSG) and reduce toxic peroxides to nontoxic hydroxyl compounds.
GSTs	Key enzymes in the GSH-binding reaction. They catalyze the initial steps of the GSH-binding reaction and are mainly present in the cytosol.
GR	An enzyme that uses NADPH to catalyze GSSG into reduced GSH.
SOD	An antioxidant metalloenzyme capable of catalyzing a disproportionation of superoxide anion radicals to oxygen and hydrogen peroxide.
Trx	A protein disulfide reductase that is itself reduced by thioredoxin reductase.
Catalase	A highly efficient enzyme that reduces H_2_O_2_ to water and oxygen with Fe at the catalytic site.
Glutamate-cysteine ligase	Converts glutamate and cysteine into *γ*-glutamylcysteine and catalyzes the first step of glutathione (GSH) biosynthesis.
Prx	A class of peroxidase widely present in prokaryotes and eukaryotes that scavenge ROS.

**Table 2:** The effects of Nrf2 activators on different CCVD.

Related disease	Agent	Effects
Hypertension	Dimethyl fumarate [66]	Downregulated the renin-angiotensin system
	Luteolin [67]	Improved NO bioavailability, reduced blood pressure
	Potato bioactive peptide [68]	Reduced renal damage
	Tilapia by-product oligopeptide [70]	Reduced endothelial damage

Atherosclerosis	Epigallocatechin gallate [75]	Prevent NF-*κ*B activation
	Z-Ligustilide [77]	Reduced atherosclerotic plaques
	*Trans*-3,5,4′-trimethoxystilbene [79]	Reduced foam cell formation, atherosclerotic plaque

MI	Ulinastatin [87]	Reduced the infarct area
	Lithium [90]	Against ventricular arrhythmias
	PTS-HP*β*CD complex [91]	Improved systolic function and adverse cardiac remodeling
	NBP [92]	Inhibited myocardial apoptosis
	Andrographolide [93]	Attenuated cardiac fibrosis

MIRI	Atorvastatin [88]	Reduced MI area
	Pinacidil [98]	Preserved heart function and myocardial ultrastructure
	Dihydroquercetin [101]	Alleviated cardiac dysfunction
	GYY4137 [102]	Reduced infarct size and cardiomyocyte apoptosis
	*α*-Lipoic acid [103]	Attenuated cardiac dysfunction
	TFCC [105]	Prevented myocardial damage
	Arctigenin [114]	Reduced infarct area and improved arrhythmia
	CNTF [183]	Attenuated death in myocardial cells

Heart failure/cardiac remodeling	Bardoxolone methyl [108]	Attenuated myocardial inflammation
	NBP [115]	Improved ventricular function and prevents ventricular remodeling
	Zingerone [116]	Suppressed cardiac hypertrophy, fibrosis, and inflammation
	Sodium sulfide [119]	Improved left ventricular function and cardiac hypertrophy
	DH404 [118]	Inhibited cardiac remodeling, dysfunction
	Nitrite [120]	Ameliorated myocardial dysfunction

Cerebral hemorrhage	RS9 [130]	Decreased brain edema, neuronal damage area, and neurological deficits
	Simvastatin [132]	Alleviated inflammatory injury, promoted neurological recovery
	Monomethyl fumarate [133]	Improved neurological deficit
	Isoliquiritigenin [129]	Alleviated neurological deficits, histological damage, and blood-brain barrier disruption
	Nicotinamide mononucleotide [136]	Promoted the recovery of neurological function
	*tert*-Butylhydroquinone [135]	Reduced oxidative brain damage
	Melatonin [134]	Ameliorated early brain injury
	Mangiferin [137]	Ameliorated their neurological deficits and brain edema
	Sulforaphane [138]	Attenuated vasospasm, ameliorated behavioral functions disrupted

CI	Urinary kallidinogenase [140]	Reduced neurological deficit and the area of CI
	Tissue kallikrein [141]	Improved neurological deficits and reduced the infarct size
	Salvianolate lyophilized injection [142]	Increased the number of brain microvasculature in ipsilateral
	Huang-Lian-Jie-Du decoction [143]	Reduced neuron structure damage, neuronal loss
	Andrographolide [144]	Suppressed blood-brain barrier disruption, and brain infarction
	BMMCs [145]	Improved the neurological function and reduced the infarct scope

CI/RI	Chlorogenic acid [149]	Reduced brain damage, enhanced learning, and spatial memory
	Lyciumamide A [150]	Ameliorated oxidative damage and neuronal apoptosis
	COM 3 [151]	Improved the neuronal mitochondrial energy metabolism
	Schizandrin A [153]	Improved the neurological score and reduced infarct volume
	Uric acid [154]	Decreased the infarct volume and neurological deficit
	AFF [155]	Reduced neurologic deficits, infarct area, and histologic changes

**Table 3:** Clinical trials of Nrf2 activators.

Name	Registration no.	Target disease	Status	Study period
Sulforaphane	NCT02614742	SAH	Phase 2	Apr. 2016-Mar. 2019
	NCT02801448	Type 2 diabetes	Phase 2	Sept. 2015-Jun. 2020
	NCT02970682	Breast neoplasm	Phase 2	Oct. 2016-Jan. 2019

Bardoxolone methyl	NCT03068130	Pulmonary hypertension	Phase 3	Apr. 2017-Dec. 2021
	NCT00811889	Chronic kidney disease/type 2 diabetes	Phase 2	Apr. 2009-May 2010
	NCT00664027	Diabetic nephropathy	Phase 2	Apr. 2008-May 2009

Tecfidera	NCT00451451	Multiple sclerosis	Launched	Jun. 2007-Aug. 2011

Epigallocatechin gallate	NCT02015312	Cardiac amyloid light-chain amyloidosis	Phase 2	Apr. 2013-Oct. 2017
	NCT01923597	Diabetic nephropathy/hypertension	Phase 2	Nov. 2013-Mar. 2015
	NCT02731495	Traumatic brain injury	Phase 2/3	Mar. 2015-Nov. 2015

Dimethyl fumarate	NCT02461069	Multiple sclerosis	Phase 4	May. 2015-Jan. 2018
	NCT02784834	Chronic lymphocytic leukemia	Phase 1	Jun. 2016-Feb. 2019
	NCT02546440	Cutaneous T cell lymphoma	Phase 2	Sept. 2015-Sept. 2020

NBP	NCT00724724	Stroke	Phase 4	Aug. 2008-Jun. 2011
	NCT02149875	Acute ischemic stroke	Phase 1/2	Jan. 2010-May 2010

Andrographolide	NCT03132623	Acute bronchitis	Phase 4	Dec. 2016-Dec. 2017
	NCT02644590	Diabetes mellitus/type 1 hypertension	Phase 1	Feb. 2016-Sept. 2016

## Abbreviations

ARE: Antioxidant response element
ATG: Arctigenin
Bach1: BTB and CNC homology 1
BMMCs: Bone marrow mononuclear cells
CCVD: Cardiocerebral vascular disease
CVD: Cardiovascular diseases
CD: Cerebrovascular disease
CI/RI: Cerebral ischemia/reperfusion injury
CTR: C-Terminal region
CNC: Cap-n-collar
CHD6: Chromo-ATPase/helicase DNA-binding protein 6
CI: Cerebral infarction
CDDO: 2-Cyano-3,12-dioxooleana-1,9-dien-28-oic acid
DGR: Double glycine repeat
ERK: Extracellular signal-regulated protein kinase
GST: Glutathione-S-transferase
GSH: Glutathione
GSK-3*β*: Glycogen synthase kinase-3*β*
GR: Glutathione reductase
GRx: Glutaredoxin
HO-1: Heme oxygenase-1
IVR: Intervening region
ICH: Intracranial hemorrhage
JNK: C-Jun N-terminal kinase
Keap1: Kelch-like ECH-associated protein 1
MAPKs: Mitogen-activated protein kinases
miRNAs: MicroRNAs
MI: Myocardial infarction
MIRI: Myocardial ischemia-reperfusion injury
MCAO: Middle cerebral artery occlusion
NTR: N-Terminal region
NADPH: Reduced form of nicotinamide adenine dinucleotide phosphate
Nrf2: NF-E2-related factor 2
NQO1: NAD(P)H dehydrogenase, quinone 1
NOS: Nitric oxide synthase
OS: Oxidative stress
NBP: 3-n-Butylphthalide
OGD/R: Oxygen glucose deprivation/reperfusion
GSSG: Oxidized glutathione
Prx: Peroxiredoxin
PI3K: Phosphatidylinositol 3-kinase
PKC: Protein kinase C
ROS: Reactive oxygen species
RNS: Reactive nitrogen species
RVLM: Rostral ventrolateral medulla
SOD: Superoxide dismutase
sMaf: Small musculoaponeurotic fibrosarcoma
SRC3: Steroid receptor coactivator 3
SAH: Subarachnoid hemorrhage
SBI: Secondary brain injury
Trx: Thioredoxin
*β*-TrCP: *β*-Transducin repeat-containing protein
VECs: Vascular endothelial cells
VSMCs: Vascular smooth muscle cells.

## References

[1] Zhang X.-f., Hu D.-y., Ding R.-j., Wang H.-c., Yan L.-x. (2012). Status and trend of cardio-cerebral-vascular diseases mortality in China: data from national disease surveillance system between 2004 and 2008. *Zhonghua Xin Xue Guan Bing Za Zhi*.

[2] Carallo C., Irace C., de Franceschi M. S. (2011). The effect of aging on blood and plasma viscosity. An 11.6 years follow-up study. *Clinical Hemorheology and Microcirculation*.

[3] Owens A. P., Byrnes J. R., Mackman N. (2014). Hyperlipidemia, tissue factor, coagulation, and simvastatin. *Trends in Cardiovascular Medicine*.

[4] Perumareddi P. (2019). Prevention of hypertension related to cardiovascular disease. *Primary Care*.

[5] Di Meo S., Reed T. T., Venditti P., Victor V. M. (2016). Role of ROS and RNS sources in physiological and pathological conditions. *Oxidative Medicine and Cellular Longevity*.

[6] Dikalov S. (2011). Cross talk between mitochondria and NADPH oxidases. *Free Radical Biology & Medicine*.

[7] Santilli F., D'Ardes D., Davì G. (2015). Oxidative stress in chronic vascular disease: from prediction to prevention. *Vascular Pharmacology*.

[8] Wu X., Zhang H., Qi W. (2018). Nicotine promotes atherosclerosis via ROS-NLRP3-mediated endothelial cell pyroptosis. *Cell Death & Disease*.

[9] Wang Z., Liu B., Zhu J., Wang D., Wang Y. (2019). Nicotine-mediated autophagy of vascular smooth muscle cell accelerates atherosclerosis via nAChRs/ROS/NF-*κ*B signaling pathway. *Atherosclerosis*.

[10] Förstermann U., Xia N., Li H. (2017). Roles of vascular oxidative stress and nitric oxide in the pathogenesis of atherosclerosis. *Circulation Research*.

[11] Eastman C. L., D'Ambrosio R., Ganesh T. (2020). Modulating neuroinflammation and oxidative stress to prevent epilepsy and improve outcomes after traumatic brain injury. *Neuropharmacology*.

[12] Kasai S., Shimizu S., Tatara Y., Mimura J., Itoh K. (2020). Regulation of Nrf2 by mitochondrial reactive oxygen species in physiology and pathology. *Biomolecules*.

[13] Li J., Ichikawa T., Janicki J. S., Cui T. (2009). Targeting the Nrf2 pathway against cardiovascular disease. *Expert Opinion on Therapeutic Targets*.

[14] Sivandzade F., Prasad S., Bhalerao A., Cucullo L. (2019). NRF2 and NF-κB interplay in cerebrovascular and neurodegenerative disorders: molecular mechanisms and possible therapeutic approaches. *Redox Biology*.

[15] Sivandzade F., Bhalerao A., Cucullo L. (2019). Cerebrovascular and Neurological Disorders: Protective Role of NRF2. *International Journal of Molecular Sciences*.

[16] Cullinan S. B., Gordan J. D., Jin J., Harper J. W., Diehl J. A. (2004). The Keap1-BTB protein is an adaptor that bridges Nrf2 to a Cul3-based E3 ligase: oxidative stress sensing by a Cul3-Keap1 ligase. *Molecular and Cellular Biology*.

[17] Nguyen T., Sherratt P. J., Huang H. C., Yang C. S., Pickett C. B. (2003). Increased protein stability as a mechanism that enhances Nrf2-mediated transcriptional activation of the antioxidant response element. Degradation of Nrf2 by the 26 S proteasome. *The Journal of Biological Chemistry*.

[18] Zipper L. M., Mulcahy R. T. (2002). The Keap1 BTB/POZ dimerization function is required to sequester Nrf2 in cytoplasm. *The Journal of Biological Chemistry*.

[19] Chauhan N., Chaunsali L., Deshmukh P., Padmanabhan B. (2013). Analysis of dimerization of BTB-IVR domains of Keap1 and its interaction with Cul3, by molecular modeling. *Bioinformation*.

[20] Komatsu M., Kurokawa H., Waguri S. (2010). The selective autophagy substrate p62 activates the stress responsive transcription factor Nrf2 through inactivation of Keap1. *Nature Cell Biology*.

[21] Wakabayashi N., Dinkova-Kostova A. T., Holtzclaw W. D. (2004). Protection against electrophile and oxidant stress by induction of the phase 2 response: fate of cysteines of the Keap1 sensor modified by inducers. *Proceedings of the National Academy of Sciences of the United States of America*.

[22] Dayalan Naidu S., Muramatsu A., Saito R. (2018). C151 in KEAP1 is the main cysteine sensor for the cyanoenone class of NRF2 activators, irrespective of molecular size or shape. *Scientific Reports*.

[23] Moi P., Chan K., Asunis I., Cao A., Kan Y. W. (1994). Isolation of NF-E2-related factor 2 (Nrf2), a NF-E2-like basic leucine zipper transcriptional activator that binds to the tandem NF-E2/AP1 repeat of the beta-globin locus control region. *Proceedings of the National Academy of Sciences of the United States of America*.

[24] Kannan M. B., Solovieva V., Blank V. (2012). The small MAF transcription factors MAFF, MAFG and MAFK: current knowledge and perspectives. *Biochimica et Biophysica Acta*.

[25] Tong K. I., Padmanabhan B., Kobayashi A. (2007). Different electrostatic potentials define ETGE and DLG motifs as hinge and latch in oxidative stress response. *Molecular and Cellular Biology*.

[26] Fukutomi T., Takagi K., Mizushima T., Ohuchi N., Yamamoto M. (2014). Kinetic, thermodynamic, and structural characterizations of the association between Nrf2-DLGex degron and Keap1. *Molecular and Cellular Biology*.

[27] Shibata T., Ohta T., Tong K. I. (2008). Cancer related mutations in NRF2 impair its recognition by Keap1-Cul3 E3 ligase and promote malignancy. *Proceedings of the National Academy of Sciences of the United States of America*.

[28] Nioi P., Nguyen T., Sherratt P. J., Pickett C. B. (2005). The carboxy-terminal Neh3 domain of Nrf2 is required for transcriptional activation. *Molecular and Cellular Biology*.

[29] Katoh Y., Itoh K., Yoshida E., Miyagishi M., Fukamizu A., Yamamoto M. (2001). Two domains of Nrf2 cooperatively bind CBP, a CREB binding protein, and synergistically activate transcription. *Genes to Cells*.

[30] Chowdhry S., Zhang Y., McMahon M., Sutherland C., Cuadrado A., Hayes J. D. (2013). Nrf2 is controlled by two distinct *β*-TrCP recognition motifs in its Neh6 domain, one of which can be modulated by GSK-3 activity. *Oncogene*.

[31] Wang H., Liu K., Geng M. (2013). RXR*α* inhibits the NRF2-ARE signaling pathway through a direct interaction with the Neh7 domain of NRF2. *Cancer Research*.

[32] Uruno A., Motohashi H. (2011). The Keap1-Nrf2 system as an *in vivo* sensor for electrophiles. *Nitric Oxide*.

[33] Baird L., Dinkova-Kostova A. T. (2013). Diffusion dynamics of the Keap1-Cullin3 interaction in single live cells. *Biochemical and Biophysical Research Communications*.

[34] Suzuki T., Yamamoto M. (2015). Molecular basis of the Keap1-Nrf2 system. *Free Radical Biology and Medicine*.

[35] Jain A., Lamark T., Sjøttem E. (2010). p62/SQSTM1 is a target gene for transcription factor NRF2 and creates a positive feedback loop by inducing antioxidant response element-driven gene transcription. *The Journal of Biological Chemistry*.

[36] Ichimura Y., Waguri S., Sou Y. S. (2013). Phosphorylation of p62 activates the Keap1-Nrf2 pathway during selective autophagy. *Molecular Cell*.

[37] Su X., Li T., Liu Z. (2018). Licochalcone A activates Keap1-Nrf2 signaling to suppress arthritis via phosphorylation of p62 at serine 349. *Free Radical Biology & Medicine*.

[38] Cuadrado A. (2015). Structural and functional characterization of Nrf2 degradation by glycogen synthase kinase 3/*β*-TrCP. *Free Radical Biology and Medicine*.

[39] Wu T., Zhao F., Gao B. (2014). Hrd1 suppresses Nrf2-mediated cellular protection during liver cirrhosis. *Genes & Development*.

[40] Yamamoto K., Suzuki N., Wada T. (2008). Human HRD1 promoter carries a functional unfolded protein response element to which XBP1 but not ATF6 directly binds. *Journal of Biochemistry*.

[41] Doroudgar S., Völkers M., Thuerauf D. J. (2015). Hrd1 and ER-associated protein degradation, ERAD, are critical elements of the adaptive ER stress response in cardiac myocytes. *Circulation Research*.

[42] Sun Z., Huang Z., Zhang D. D. (2009). Phosphorylation of Nrf2 at multiple sites by MAP kinases has a limited contribution in modulating the Nrf2-dependent antioxidant response. *Plos One*.

[43] Keum Y. S., Owuor E. D., Kim B. R., Hu R., Kong A. N. T. (2003). Involvement of Nrf2 and JNK1 in the activation of antioxidant responsive element (ARE) by chemopreventive agent phenethyl isothiocyanate (PEITC). *Pharmaceutical Research*.

[44] Alam J., Wicks C., Stewart D. (2000). Mechanism of heme oxygenase-1 gene activation by cadmium in MCF-7 mammary epithelial cells. Role of OF p38 kinase and Nrf2 transcription factor. *The Journal of Biological Chemistry*.

[45] Salazar M., Rojo A. I., Velasco D., de Sagarra R. M., Cuadrado A. (2006). Glycogen synthase kinase-3beta inhibits the xenobiotic and antioxidant cell response by direct phosphorylation and nuclear exclusion of the transcription factor Nrf2. *The Journal of Biological Chemistry*.

[46] Shang G., Tang X., Gao P. (2015). Sulforaphane attenuation of experimental diabetic nephropathy involves GSK-3 beta/Fyn/Nrf2 signaling pathway. *The Journal of Nutritional Biochemistry*.

[47] Bushati N., Cohen S. M. (2007). MicroRNA functions. *Annual Review of Cell and Developmental Biology*.

[48] Narasimhan M., Patel D., Vedpathak D., Rathinam M., Henderson G., Mahimainathan L. (2012). Identification of novel microRNAs in post-transcriptional control of Nrf2 expression and redox homeostasis in neuronal, SH-SY5Y cells. *PLoS One*.

[49] Li F., Liang J., Tong H., Zhu S., Tang D. (2020). Inhibition of microRNA-199a-5p ameliorates oxygen-glucose deprivation/reoxygenation-induced apoptosis and oxidative stress in HT22 neurons by targeting Brg1 to activate Nrf2/HO-1 signalling. *Clinical and Experimental Pharmacology & Physiology*.

[50] Weng C. F., Wu C. F., Kao S. H., Chen J. C., Lin H. H. (2019). Down-regulation of miR-34a-5p potentiates protective effect of adipose-derived mesenchymal stem cells against ischemic myocardial infarction by stimulating the expression of C1q/tumor necrosis factor-related protein-9. *Frontiers in Physiology*.

[51] Sun J., Brand M., Zenke Y., Tashiro S., Groudine M., Igarashi K. (2004). Heme regulates the dynamic exchange of Bach1 and NF-E2-related factors in the Maf transcription factor network. *Proceedings of the National Academy of Sciences of the United States of America*.

[52] Banning A., Brigelius-Flohe R. (2005). NF-kappaB, Nrf2, and HO-1 interplay in redox-regulated VCAM-1 expression. *Antioxidants & Redox Signaling*.

[53] Wardyn J. D., Ponsford A. H., Sanderson C. M. (2015). Dissecting molecular cross-talk between Nrf2 and NF-*κ*B response pathways. *Biochemical Society Transactions*.

[54] Cuadrado A., Martín-Moldes Z., Ye J., Lastres-Becker I. (2014). Transcription factors NRF2 and NF-*κ*B are coordinated effectors of the rho family, GTP-binding protein RAC1 during inflammation. *The Journal of Biological Chemistry*.

[55] Siebel C., Lendahl U. (2017). Notch signaling in development, tissue homeostasis, and disease. *Physiological Reviews*.

[56] Wakabayashi N., Skoko J. J., Chartoumpekis D. V. (2014). Notch-Nrf2 axis: regulation of Nrf2 gene expression and cytoprotection by notch signaling. *Molecular and Cellular Biology*.

[57] Paul M. K., Bisht B., Darmawan D. O. (2014). Dynamic changes in intracellular ROS levels regulate airway basal stem cell homeostasis through Nrf2-dependent notch signaling. *Cell Stem Cell*.

[58] Jin Y., Liu W., Liu X. (2018). Transplantation of endothelial progenitor cells attenuated paraquat-induced acute lung injury via miR-141-3p-notch-Nrf2 axis. *Cell & Bioscience*.

[59] Naidu S. D., Kostov R. V., Dinkova-Kostova A. T. (2015). Transcription factors Hsf1 and Nrf2 engage in crosstalk for cytoprotection. *Trends in Pharmacological Sciences*.

[60] Senoner T., Dichtl W. (2019). Oxidative stress in cardiovascular diseases: still a therapeutic target?. *Nutrients*.

[61] Tsutsui H., Kinugawa S., Matsushima S. (2011). Oxidative stress and heart failure. *American Journal of Physiology. Heart and Circulatory Physiology*.

[62] da Costa R. M., Rodrigues D., Pereira C. A. (2019). Nrf2 as a potential mediator of cardiovascular risk in metabolic diseases. *Frontiers in Pharmacology*.

[63] Barančík M., Grešová L., Barteková M., Dovinová I. (2016). Nrf2 as a key player of redox regulation in cardiovascular diseases. *Physiological Research*.

[64] Smyrnias I., Zhang X., Zhang M. (2015). Nicotinamide adenine dinucleotide phosphate oxidase-4-dependent upregulation of nuclear factor erythroid-derived 2-like 2 protects the heart during chronic pressure overload. *Hypertension*.

[65] Silva-Palacios A., Königsberg M., Zazueta C. (2016). Nrf2 signaling and redox homeostasis in the aging heart: a potential target to prevent cardiovascular diseases?. *Ageing Research Reviews*.

[66] Hsu C.-N., Lin Y.-J., Yu H.-R. (2019). Protection of male rat offspring against hypertension programmed by prenatal dexamethasone administration and postnatal high-fat diet with the Nrf2 activator dimethyl fumarate during pregnancy. *International Journal of Molecular Sciences*.

[67] Oyagbemi A. A., Omobowale T. O., Ola-Davies O. E. (2018). Luteolin-mediated Kim-1/NF-kB/Nrf2 signaling pathways protects sodium fluoride-induced hypertension and cardiovascular complications. *BioFactors*.

[68] Tsai B. C.-K., Hsieh D. J.-Y., Lin W.-T. (2020). Functional potato bioactive peptide intensifies Nrf2-dependent antioxidant defense against renal damage in hypertensive rats. *Food Research International*.

[69] Yang Y., Tian T., Wang Y., Li Z., Xing K., Tian G. (2019). SIRT6 protects vascular endothelial cells from angiotensin II-induced apoptosis and oxidative stress by promoting the activation of Nrf2/ARE signaling. *European Journal of Pharmacology*.

[70] Chen J., Gong F., Chen M.-F. (2019). In vitro vascular-protective effects of a tilapia by-product oligopeptide on angiotensin II-induced hypertensive endothelial injury in HUVEC by Nrf2/NF-*κ*B pathways. *Marine Drugs*.

[71] Gao L., Zimmerman M. C., Biswal S., Zucker I. H. (2017). Selective Nrf2 gene deletion in the rostral ventrolateral medulla evokes hypertension and sympathoexcitation in mice. *Hypertension*.

[72] Sarutipaiboon I., Settasatian N., Komanasin N. (2020). Association of genetic variations in NRF2, NQO1, HMOX1, and MT with severity of coronary artery disease and related risk factors. *Cardiovascular Toxicology*.

[73] Huppke P., Weissbach S., Church J. A. (2017). Activating de novo mutations in *NFE2L2* encoding NRF2 cause a multisystem disorder. *Nature Communications*.

[74] Ruotsalainen A. K., Inkala M., Partanen M. E. (2013). The absence of macrophage Nrf2 promotes early atherogenesis. *Cardiovascular Research*.

[75] Yamagata K. (2020). Protective effect of epigallocatechin gallate on endothelial disorders in atherosclerosis. *Journal of Cardiovascular Pharmacology*.

[76] Jiang J., Mo Z. C., Yin K. (2012). Epigallocatechin-3-gallate prevents TNF-*α*-induced NF-*κ*B activation thereby upregulating ABCA1 via the Nrf2/Keap1 pathway in macrophage foam cells. *International Journal of Molecular Medicine*.

[77] Zhu Y., Zhang Y., Huang X. (2019). Z-Ligustilide protects vascular endothelial cells from oxidative stress and rescues high fat diet-induced atherosclerosis by activating multiple *NRF2* downstream genes. *Atherosclerosis*.

[78] Seo Y., Park J., Choi W. (2019). Antiatherogenic effect of resveratrol attributed to decreased expression of ICAM-1 (intercellular adhesion molecule-1). *Arteriosclerosis, Thrombosis, and Vascular Biology*.

[79] Hong M., Li J., Li S., Almutairi M. M. (2020). Resveratrol Derivative,Trans‐3, 5, 4′-trimethoxystilbene, prevents the developing of atherosclerotic lesions and attenuates cholesterol accumulation in macrophage foam cells. *Molecular Nutrition & Food Research*.

[80] Barajas B., Che N., Yin F. (2011). NF-E2-related factor 2 promotes atherosclerosis by effects on plasma lipoproteins and cholesterol transport that overshadow antioxidant protection. *Arteriosclerosis, Thrombosis, and Vascular Biology*.

[81] Sussan T. E., Jun J., Thimmulappa R. (2008). Disruption of Nrf2, a key inducer of antioxidant defenses, attenuates ApoE-mediated atherosclerosis in mice. *PLoS One*.

[82] Sawada H., Saito Y., Noguchi N. (2012). Enhanced *CD36* expression changes the role of Nrf2 activation from anti- atherogenic to pro-atherogenic in apoE-deficient mice. *Atherosclerosis*.

[83] Freigang S., Ampenberger F., Spohn G. (2011). Nrf2 is essential for cholesterol crystal-induced inflammasome activation and exacerbation of atherosclerosis. *European Journal of Immunology*.

[84] Mimura J., Itoh K. (2015). Role of Nrf2 in the pathogenesis of atherosclerosis. *Free Radical Biology and Medicine*.

[85] Strom J., Chen Q. M. (2017). Loss of Nrf2 promotes rapid progression to heart failure following myocardial infarction. *Toxicology and Applied Pharmacology*.

[86] Liberale (2019). IL-1*β* and statin treatment in patients with myocardial infarction and diabetic cardiomyopathy. *Journal of Clinical Medicine*.

[87] Wang S., Cheng Z. Y., Chen X. J., Xue H. Z. (2018). Ulinastatin protects rats with myocardial infarction by activating Nrf2/NOS pathway. *European Review for Medical and Pharmacological Sciences*.

[88] Sun G., Li Y., Ji Z. (2015). Atorvastatin attenuates inflammation and oxidative stress induced by ischemia/reperfusion in rat heart via the Nrf2 transcription factor. *International Journal of Clinical and Experimental Medicine*.

[89] Yeh Y. H., Kuo C. T., Chang G. J. (2015). Rosuvastatin suppresses atrial tachycardia-induced cellular remodeling via Akt/Nrf2/heme oxygenase-1 pathway. *Journal of Molecular and Cellular Cardiology*.

[90] Lee T. M., Lin S. Z., Chang N. C. (2014). Antiarrhythmic effect of lithium in rats after myocardial infarction by activation of Nrf2/HO-1 signaling. *Free Radical Biology & Medicine*.

[91] Lacerda D., Ortiz V., Türck P. (2018). Stilbenoid pterostilbene complexed with cyclodextrin preserves left ventricular function after myocardial infarction in rats: possible involvement of thiol proteins and modulation of phosphorylated GSK-3*β*. *Free Radical Research*.

[92] Bai M., Pan C. L., Jiang G. X., Zhang Y. M., Zhang Z. (2019). Effects of butylphthalide on oxidative stress and inflammatory response in rats with myocardial infarction through Akt/Nrf2 signaling pathway. *European Review for Medical and Pharmacological Sciences*.

[93] Xie S., Deng W., Chen J. (2020). Andrographolide protects against adverse cardiac remodeling after myocardial infarction through enhancing Nrf2 signaling pathway. *International Journal of Biological Sciences*.

[94] Shen Y., Liu X., Shi J., Wu X. (2019). Involvement of Nrf2 in myocardial ischemia and reperfusion injury. *International Journal of Biological Macromolecules*.

[95] Giordano F. J. (2005). Oxygen, oxidative stress, hypoxia, and heart failure. *The Journal of Clinical Investigation*.

[96] Cadenas S. (2018). ROS and redox signaling in myocardial ischemia-reperfusion injury and cardioprotection. *Free Radical Biology & Medicine*.

[97] Xu B., Zhang J., Strom J., Lee S., Chen Q. M. (2014). Myocardial ischemic reperfusion induces de novo Nrf2 protein translation. *Biochimica et Biophysica Acta*.

[98] Yang Y. H., Zhang Y., Chen W. (2016). Pinacidil-postconditioning is equivalent to ischemic postconditioning in defeating cardiac ischemia-reperfusion injury in rat. *European Journal of Pharmacology*.

[99] Yin X., Wang X., Fan Z. (2015). Hyperbaric oxygen preconditioning attenuates myocardium ischemia-reperfusion injury through upregulation of heme oxygenase 1 expression: PI3K/Akt/Nrf2 pathway involved. *Journal of Cardiovascular Pharmacology and Therapeutics*.

[100] Li M., Xu S., Geng Y. (2019). The protective effects of L-carnitine on myocardial ischaemia-reperfusion injury in patients with rheumatic valvular heart disease undergoingCPBsurgery are associated with the suppression ofNF‐*κ*B pathway and the activation of Nrf2 pathway. *Clinical and Experimental Pharmacology & Physiology*.

[101] Shu Z., Yang Y., Yang L., Jiang H., Yu X., Wang Y. (2019). Cardioprotective effects of dihydroquercetin against ischemia reperfusion injury by inhibiting oxidative stress and endoplasmic reticulum stress-induced apoptosisviathe PI3K/Akt pathway. *Food & Function*.

[102] Qiu Y., Wu Y., Meng M. (2018). GYY4137 protects against myocardial ischemia/reperfusion injury via activation of the PHLPP-1/Akt/Nrf2 signaling pathway in diabetic mice. *The Journal of Surgical Research*.

[103] Deng C., Sun Z., Tong G. (2013). *α*-Lipoic acid reduces infarct size and preserves cardiac function in rat myocardial ischemia/reperfusion injury through activation of PI3K/Akt/Nrf2 pathway. *PLoS One*.

[104] Ding S., Yang Y., Mei J. (2016). Protective effects of L-malate against myocardial ischemia/reperfusion injury in rats. *Evidence-based Complementary and Alternative Medicine*.

[105] Zhang H. J., Chen R. C., Sun G. B. (2018). Protective effects of total flavonoids from Clinopodium chinense (Benth.) O. Ktze on myocardial injury in vivo and in vitro via regulation of Akt/Nrf2/HO-1 pathway. *Phytomedicine*.

[106] Liao H.-H., Zhang N., Meng Y.-y. (2019). Myricetin alleviates pathological cardiac hypertrophy via TRAF6/TAK1/MAPK and Nrf2 signaling pathway. *Oxidative Medicine and Cellular Longevity*.

[107] Zhao G. J., Hou N., Cai S. A. (2018). Contributions of Nrf2 to puerarin prevention of cardiac hypertrophy and its metabolic enzymes expression in rats. *The Journal of Pharmacology and Experimental Therapeutics*.

[108] Tian C., Gao L., Zhang A., Hackfort B. T., Zucker I. H. (2019). Therapeutic effects of Nrf2 activation by bardoxolone methyl in chronic heart failure. *The Journal of Pharmacology and Experimental Therapeutics*.

[109] Ma A., Hong J., Shanks J. (2019). Upregulating Nrf2 in the RVLM ameliorates sympatho-excitation in mice with chronic heart failure. *Free Radical Biology & Medicine*.

[110] Wafi A. M., Yu L., Gao L., Zucker I. H. (2019). Exercise training upregulates Nrf2 protein in the rostral ventrolateral medulla of mice with heart failure. *Journal of Applied Physiology*.

[111] Li J., Zhang C., Xing Y. (2011). Up-regulation of p27(kip1) contributes to Nrf2-mediated protection against angiotensin II-induced cardiac hypertrophy. *Cardiovascular Research*.

[112] Xin Y., Bai Y., Jiang X. (2018). Sulforaphane prevents angiotensin II-induced cardiomyopathy by activation of Nrf2 via stimulating the Akt/GSK-3ß/Fyn pathway. *Redox Biology*.

[113] Chen R. R., Fan X. H., Chen G. (2019). Irisin attenuates angiotensin II-induced cardiac fibrosis *via* Nrf2 mediated inhibition of ROS/ TGF *β*1/Smad2/3 signaling axis. *Chemico-Biological Interactions*.

[114] Yang J., Yin H. S., Cao Y. J. (2018). Arctigenin attenuates ischemia/reperfusion induced ventricular arrhythmias by decreasing oxidative stress in rats. *Cellular Physiology and Biochemistry*.

[115] Qiu H., Ma J., Wu H., Ding C. (2018). DL-3-n-Butylphthalide improves ventricular function, and prevents ventricular remodeling and arrhythmias in post-MI rats. *Naunyn-Schmiedeberg's Archives of Pharmacology*.

[116] Liu C., Wu Q. Q., Cai Z. L. (2019). Zingerone attenuates aortic banding-induced cardiac remodelling via activating the eNOS/Nrf2 pathway. *Journal of Cellular and Molecular Medicine*.

[117] Xing Y., Niu T., Wang W. (2012). Triterpenoid dihydro-CDDO-trifluoroethyl amide protects against maladaptive cardiac remodeling and dysfunction in mice: a critical role of Nrf2. *PLoS One*.

[118] Ichikawa T., Li J., Meyer C. J., Janicki J. S., Hannink M., Cui T. (2009). Dihydro-CDDO-trifluoroethyl amide (dh404), a novel Nrf2 activator, suppresses oxidative stress in cardiomyocytes. *PLoS One*.

[119] Shimizu Y., Nicholson C. K., Lambert J. P. (2016). Sodium sulfide attenuates ischemic-induced heart failure by enhancing proteasomal function in an Nrf2-dependent manner. *Circulation. Heart Failure*.

[120] Donnarumma E., Bhushan S., Bradley J. M. (2016). Nitrite therapy ameliorates myocardial dysfunction via H2S and nuclear factor-erythroid 2-related factor 2 (Nrf2)-dependent signaling in chronic heart failure. *Journal of the American Heart Association*.

[121] Jung J. E., Kim G. S., Chen H. (2010). Reperfusion and neurovascular dysfunction in stroke: from basic mechanisms to potential strategies for neuroprotection. *Molecular Neurobiology*.

[122] Lakhan S. E., Kirchgessner A., Hofer M. (2009). Inflammatory mechanisms in ischemic stroke: therapeutic approaches. *Journal of Translational Medicine*.

[123] Candelario-Jalil E. (2009). Injury and repair mechanisms in ischemic stroke: considerations for the development of novel neurotherapeutics. *Current Opinion in Investigational Drugs*.

[124] Das A., Gopalakrishnan B., Voss O. H., Doseff A. I., Villamena F. A. (2012). Inhibition of ROS-induced apoptosis in endothelial cells by nitrone spin traps *via* induction of phase II enzymes and suppression of mitochondria-dependent pro-apoptotic signaling. *Biochemical Pharmacology*.

[125] Fallenius M., Skrifvars M. B., Reinikainen M. (2019). Spontaneous intracerebral hemorrhage. *Stroke*.

[126] Zolnourian A., Galea I., Bulters D. (2019). Neuroprotective role of the Nrf2 pathway in subarachnoid haemorrhage and its therapeutic potential. *Oxidative Medicine and Cellular Longevity*.

[127] Vargas M. R., Johnson J. A. (2009). The Nrf2-ARE cytoprotective pathway in astrocytes. *Expert Reviews in Molecular Medicine*.

[128] Lee J. M., Shih A. Y., Murphy T. H., Johnson J. A. (2003). NF-E2-related factor-2 mediates neuroprotection against mitochondrial complex I inhibitors and increased concentrations of intracellular calcium in primary cortical neurons. *The Journal of Biological Chemistry*.

[129] Zeng J., Chen Y., Ding R. (2017). Isoliquiritigenin alleviates early brain injury after experimental intracerebral hemorrhage via suppressing ROS- and/or NF-*κ*B-mediated NLRP3 inflammasome activation by promoting Nrf2 antioxidant pathway. *Journal of Neuroinflammation*.

[130] Sugiyama T., Imai T., Nakamura S. (2018). A novel Nrf2 activator, RS9, attenuates secondary brain injury after intracerebral hemorrhage in sub-acute phase. *Brain Research*.

[131] Liu X. C., Wu C. Z., Hu X. F. (2020). Gastrodin attenuates neuronal apoptosis and neurological deficits after experimental intracerebral hemorrhage. *Journal of Stroke and Cerebrovascular Diseases*.

[132] Zhang C. Y., Ren X. M., Li H. B. (2019). Simvastatin alleviates inflammation and oxidative stress in rats with cerebral hemorrhage through Nrf2-ARE signaling pathway. *European Review for Medical and Pharmacological Sciences*.

[133] Shi Y. Y., Cui H. F., Qin B. J. (2019). Monomethyl fumarate protects cerebral hemorrhage injury in rats via activating microRNA-139/Nrf2 axis. *European Review for Medical and Pharmacological Sciences*.

[134] Wang Z., Ma C., Meng C. J. (2012). Melatonin activates the Nrf2-ARE pathway when it protects against early brain injury in a subarachnoid hemorrhage model. *Journal of Pineal Research*.

[135] Sukumari-Ramesh S., Alleyne C. H. (2016). Post-injury administration of tert-butylhydroquinone attenuates acute neurological injury after intracerebral hemorrhage in mice. *Journal of Molecular Neuroscience*.

[136] Wei C. C., Kong Y. Y., Li G. Q., Guan Y. F., Wang P., Miao C. Y. (2017). Nicotinamide mononucleotide attenuates brain injury after intracerebral hemorrhage by activating Nrf2/HO-1 signaling pathway. *Scientific Reports*.

[137] Wang Z., Guo S., Wang J., Shen Y., Zhang J., Wu Q. (2017). Nrf2/HO-1 mediates the neuroprotective effect of mangiferin on early brain injury after subarachnoid hemorrhage by attenuating mitochondria-related apoptosis and neuroinflammation. *Scientific Reports*.

[138] Zhao X., Wen L., Dong M., Lu X. (2016). Sulforaphane activates the cerebral vascular Nrf2-ARE pathway and suppresses inflammation to attenuate cerebral vasospasm in rat with subarachnoid hemorrhage. *Brain Research*.

[139] Bereczki D., Balla J., Bereczki D. (2018). Heme oxygenase-1: clinical relevance in ischemic stroke. *Current Pharmaceutical Design*.

[140] Fan Q. Y., Qiu Z., Zhang X. D. (2019). Influences of urinary kallidinogenase on neuronal apoptosis in cerebral infarction rats through Nrf2/ARE oxidative stress pathway. *European Review for Medical and Pharmacological Sciences*.

[141] Yang J., Su J., Wan F. (2017). Tissue kallikrein protects against ischemic stroke by suppressing TLR4/NF-*κ*B and activating Nrf2 signaling pathway in rats. *Experimental and Therapeutic Medicine*.

[142] Wang F., He Q., Wang J. (2017). Neuroprotective effect of salvianolate lyophilized injection against cerebral ischemia in type 1 diabetic rats. *BMC Complementary and Alternative Medicine*.

[143] Zhang Q., Wang J., Zhang C. (2016). The components of Huang-Lian-Jie-Du-decoction act synergistically to exert protective effects in a rat ischemic stroke model. *Oncotarget*.

[144] Yen T. L., Chen R. J., Jayakumar T. (2016). Andrographolide stimulates p38 mitogen-activated protein kinase-nuclear factor erythroid-2-related factor 2-heme oxygenase 1 signaling in primary cerebral endothelial cells for definite protection against ischemic stroke in rats. *Translational Research*.

[145] Chen N.-N., Wang J.-P., Liu H.-F. (2017). The bone marrow mononuclear cells reduce the oxidative stress of cerebral infarction through PI3K/AKT/NRF2 signaling pathway. *European Review for Medical and Pharmacological Sciences*.

[146] Granger D. N., Kvietys P. R. (2015). Reperfusion injury and reactive oxygen species: the evolution of a concept. *Redox Biology*.

[147] Alfieri A., Srivastava S., Siow R. C. M., Modo M., Fraser P. A., Mann G. E. (2011). Targeting the Nrf2-Keap1 antioxidant defence pathway for neurovascular protection in stroke. *The Journal of Physiology*.

[148] Peng L., Zhao Y., Li Y. (2019). Effect of DJ-1 on the neuroprotection of astrocytes subjected to cerebral ischemia/reperfusion injury. *Journal of Molecular Medicine*.

[149] Liu D., Wang H., Zhang Y., Zhang Z. (2020). Protective effects of Chlorogenic acid on cerebral ischemia/reperfusion injury rats by regulating oxidative stress-related Nrf2 pathway. *Drug Design, Development and Therapy*.

[150] Gao K., Liu M., Ding Y. (2019). A phenolic amide (LyA) isolated from the fruits of *Lycium barbarum* protects against cerebral ischemia–reperfusion injury via PKCε/Nrf2/HO-1 pathway. *Aging*.

[151] Wang J., Zhang W., Lv C. (2020). A novel biscoumarin compound ameliorates cerebral ischemia reperfusion-induced mitochondrial oxidative injury via Nrf2/Keap1/ARE signaling. *Neuropharmacology*.

[152] Liu Q., Jin Z., Xu Z. (2019). Antioxidant effects of ginkgolides and bilobalide against cerebral ischemia injury by activating the Akt/Nrf2 pathway in vitro and in vivo. *Cell Stress & Chaperones*.

[153] Zhou F., Wang M., Ju J. (2019). Schizandrin A protects against cerebral ischemia-reperfusion injury by suppressing inflammation and oxidative stress and regulating the AMPK/Nrf2 pathway regulation. *American Journal of Translational Research*.

[154] Ya B.-l., Liu Q., Li H.-f. (2018). Uric acid protects against focal cerebral ischemia/reperfusion-induced oxidative stress via activating Nrf2 and regulating neurotrophic factor expression. *Oxidative Medicine and Cellular Longevity*.

[155] Luo Y., Cui H.-X., Jia A., Jia S.-S., Yuan K. (2018). The protective effect of the total flavonoids of Abelmoschus esculentus L. flowers on transient cerebral ischemia-reperfusion injury is due to activation of the Nrf2-ARE pathway. *Oxidative Medicine and Cellular Longevity*.

[156] Hu L., Chen W., Tian F., Yuan C., Wang H., Yue H. (2018). Neuroprotective role of fucoxanthin against cerebral ischemic/reperfusion injury through activation of Nrf2/HO-1 signaling. *Biomedicine & Pharmacotherapy*.

[157] Krüger-Genge (2019). Vascular endothelial cell biology: an update. *International Journal of Molecular Sciences*.

[158] Ramprasath T., Vasudevan V., Sasikumar S., Puhari S., Saso L., Selvam G. (2015). Regression of oxidative stress by targeting eNOS and Nrf2/ARE signaling: a guided drug target for cardiovascular diseases. *Current Topics in Medicinal Chemistry*.

[159] Jaminon A., Reesink K., Kroon A., Schurgers L. (2019). The role of vascular smooth muscle cells in arterial remodeling: focus on calcification-related processes. *International Journal of Molecular Sciences*.

[160] Durgin B. G., Straub A. C. (2018). Redox control of vascular smooth muscle cell function and plasticity. *Laboratory Investigation*.

[161] Aghagolzadeh P., Radpour R., Bachtler M. (2017). Hydrogen sulfide attenuates calcification of vascular smooth muscle cells via KEAP1/NRF2/NQO1 activation. *Atherosclerosis*.

[162] Yao L., Wang J., Tian B. Y., Xu T. H., Sheng Z. T. (2017). Activation of the Nrf2-ARE signaling pathway prevents hyperphosphatemia-induced vascular calcification by inducing autophagy in renal vascular smooth muscle cells. *Journal of Cellular Biochemistry*.

[163] Ashino T., Yamamoto M., Numazawa S. (2016). Nrf2/Keap1 system regulates vascular smooth muscle cell apoptosis for vascular homeostasis: role in neointimal formation after vascular injury. *Scientific Reports*.

[164] Zhao J., Niu X., Yu J. (2020). *Poria cocos* polysaccharides attenuated ox-LDL-induced inflammation and oxidative stress via ERK activated Nrf2/HO-1 signaling pathway and inhibited foam cell formation in VSMCs. *International Immunopharmacology*.

[165] Xie L., Gu Y., Wen M. (2016). Hydrogen sulfide induces Keap1 S-sulfhydration and suppresses diabetes-accelerated atherosclerosis via Nrf2 activation. *Diabetes*.

[166] Wolf S. A., Boddeke H. W. G. M., Kettenmann H. (2017). Microglia in physiology and disease. *Annual Review of Physiology*.

[167] Yu H., Wang X., Kang F., Chen Z., Meng Y., Dai M. (2019). Propofol attenuates inflammatory damage on neurons following cerebral infarction by inhibiting excessive activation of microglia. *International Journal of Molecular Medicine*.

[168] Xu L., He D., Bai Y. (2016). Microglia-mediated inflammation and neurodegenerative disease. *Molecular Neurobiology*.

[169] Lambertsen K. L., Biber K., Finsen B. (2012). Inflammatory cytokines in experimental and human stroke. *Journal of Cerebral Blood Flow and Metabolism*.

[170] Wang Y., Huang Y., Xu Y. (2018). A dual AMPK/Nrf2 activator reduces brain inflammation after stroke by enhancing microglia M2 polarization. *Antioxidants & Redox Signaling*.

[171] Chang C. Y., Kuan Y. H., Li J. R. (2013). Docosahexaenoic acid reduces cellular inflammatory response following permanent focal cerebral ischemia in rats. *The Journal of Nutritional Biochemistry*.

[172] Wang G., Wang L., Sun X. G., Tang J. (2018). Haematoma scavenging in intracerebral haemorrhage: from mechanisms to the clinic. *Journal of Cellular and Molecular Medicine*.

[173] Cheng Y., Yang C., Luo D., Li X., le X. C., Rong J. (2018). N-Propargyl caffeamide skews macrophages towards a resolving M2-like phenotype against myocardial ischemic injury via activating Nrf2/HO-1 pathway and inhibiting NF-ĸB pathway. *Cellular Physiology and Biochemistry*.

[174] Cheng Y., Tse H. F., Li X., Han Y., Rong J. (2016). Gallic acid-l-leucine (GAL) conjugate enhances macrophage phagocytosis via inducing leukotriene B4 12-hydroxydehydrogenase (LTB4DH) expression. *Molecular Immunology*.

[175] Forlenza O. V., De-Paula V. J. R., Diniz B. S. O. (2014). Neuroprotective effects of lithium: implications for the treatment of Alzheimer's disease and related neurodegenerative disorders. *ACS Chemical Neuroscience*.

[176] Akbari G. (2020). Role of zinc supplementation on ischemia/reperfusion injury in various organs. *Biological Trace Element Research*.

[177] Du Y., Ma X., Ma L. (2020). Inhibition of microRNA-148b-3p alleviates oxygen-glucose deprivation/reoxygenation-induced apoptosis and oxidative stress in HT22 hippocampal neuron via reinforcing Sestrin2/Nrf2 signalling. *Clinical and Experimental Pharmacology & Physiology*.

[178] Hou W., Zhu X., Liu J., Map J. (2020). Inhibition of miR-153 ameliorates ischemia/reperfusion-induced cardiomyocytes apoptosis by regulating Nrf2/HO-1 signaling in rats. *Biomedical Engineering Online*.

[179] Shao D., Lian Z., Di Y. (2018). Dietary compounds have potential in controlling atherosclerosis by modulating macrophage cholesterol metabolism and inflammation via miRNA. *NPJ Science of Food*.

[180] He J., Zhang R., Shao M. (2019). Efficacy and safety of low-dose IL-2 in the treatment of systemic lupus erythematosus: a randomised, double-blind, placebo-controlled trial. *Annals of the Rheumatic Diseases*.

[181] Ma J., Jin G. (2019). Epidermal growth factor protects against myocardial ischaemia reperfusion injury through activating Nrf2 signalling pathway. *Free Radical Research*.

[182] Fang Y., Zhao Y., He S. (2018). Overexpression of FGF19 alleviates hypoxia/reoxygenation-induced injury of cardiomyocytes by regulating GSK-3*β*/Nrf2/ARE signaling. *Biochemical and Biophysical Research Communications*.

[183] Zheng K., Zhang Q., Sheng Z., Li Y., Lu H. H. (2018). Ciliary neurotrophic factor (CNTF) protects myocardial cells from oxygen glucose deprivation (OGD)/re-oxygenation via activation of Akt-Nrf2 signaling. *Cellular Physiology and Biochemistry*.

[184] Wang S., Ma F., Huang L. (2018). Dl-3-n-Butylphthalide (NBP): a promising therapeutic agent for ischemic stroke. *CNS & Neurological Disorders Drug Targets*.

[185] Satoh T., Lipton S. (2017). Recent advances in understanding NRF2 as a druggable target: development of pro-electrophilic and non-covalent NRF2 activators to overcome systemic side effects of electrophilic drugs like dimethyl fumarate. *F1000Research*.

[186] Chang J., Zhang R.-M., Zhang Y. (2008). Andrographolide drop-pill in treatment of acute upper respiratory tract infection with external wind-heat syndrome: a multicenter and randomized controlled trial. *Zhong Xi Yi Jie He Xue Bao*.

[187] Straniero S., Laskar A., Savva C., Härdfeldt J., Angelin B., Rudling M. (2020). Of mice and men: murine bile acids explain species differences in the regulation of bile acid and cholesterol metabolism. *Journal of Lipid Research*.

[188] Schwartz S. M., Galis Z. S., Rosenfeld M. E., Falk E. (2007). Plaque rupture in humans and mice. *Arteriosclerosis, Thrombosis, and Vascular Biology*.

